# Clinician preference instrumental variable analysis of the effectiveness of magnesium supplementation for atrial fibrillation prophylaxis in critical care

**DOI:** 10.1038/s41598-022-21286-1

**Published:** 2022-10-19

**Authors:** Matthew G. Wilson, Aasiyah Rashan, Roman Klapaukh, Folkert W. Asselbergs, Steve K. Harris

**Affiliations:** 1 Institute of Health Informatics, Faculty of Population Health Sciences, University College London, 222 Euston Road, London, NW1 2DA UK; 2 Research Software Development Group, Research IT Services, University College London, London, UK; 3 Institute of Cardiovascular Science and Institute of Health Informatics, Faculty of Population Health Sciences, University College London, London, UK; 4 Department of Cardiology, Division of Heart & Lungs, University Medical Centre Utrecht, Utrecht University, Utrecht, The Netherlands; 5 Critical Care Department, University College London Hospitals NHS Foundation Trust, London, UK; 6 Institute of Health Informatics, University College London, London, UK

**Keywords:** Atrial fibrillation, Atrial fibrillation, Outcomes research, Translational research

## Abstract

Atrial fibrillation is a frequently encountered condition in critical illness and causes adverse effects including haemodynamic decompensation, stroke and prolonged hospital stay. It is a common practice in critical care to supplement serum magnesium for the purpose of preventing episodes of atrial fibrillation. However, no randomised studies support this practice in the non-cardiac surgery critical care population, and the effectiveness of magnesium supplementation is unclear. We sought to investigate the effectiveness of magnesium supplementation in preventing the onset of atrial fibrillation in a mixed critical care population. We conducted a single centre retrospective observational study of adult critical care patients. We utilised a natural experiment design, using the supplementation preference of the bedside critical care nurse as an instrumental variable. Using routinely collected electronic patient data, magnesium supplementation opportunities were defined and linked to the bedside nurse. Nurse preference for administering magnesium was obtained using multilevel modelling. The results were used to define "liberal" and "restrictive" supplementation groups, which were inputted into an instrumental variable regression to obtain an estimate of the effect of magnesium supplementation. 9114 magnesium supplementation opportunities were analysed, representing 2137 critical care admissions for 1914 patients. There was significant variation in magnesium supplementation practices attributable to the individual nurse, after accounting for covariates. The instrumental variable analysis showed magnesium supplementation was associated with a 3% decreased relative risk of experiencing an atrial fibrillation event (95% CI − 0.06 to − 0.004, *p* = 0.03). This study supports the strategy of routine supplementation, but further work is required to identify optimal serum magnesium targets for atrial fibrillation prophylaxis.

## Introduction

New onset Atrial Fibrillation (AF) is a common accompaniment to critical illness. Together with pre-existing AF, it is observed in nearly one-third of patients passing through the critical care unit^1^. The incidence of AF is highest following cardiac surgery (25–50%), major thoracic surgery and oesophagectomy (10–30%), and is less common following extra-thoracic surgery (11%)^2–6^. In contrast, medical critical care patients have a general incidence of 11%, with high-risk groups (e.g. septic shock) exhibiting an incidence of 50% in one study^7^.

AF arises from disturbance to the balance of pro-arrhythmogenic factors and opposing compensatory mechanisms. Bosch et al. have described in detail the physiological processes underpinning the development of "arrhythmogenic atria" in the context of critical illness. Changes to electrolyte concentration at the cardiac myocyte can often provide the trigger to atria which have been "primed" to become arrhythmic^1^.

Magnesium (Mg) plays a key role in regulating the cardiac action potential, through its action as a co-factor at the sodium/potassium ATPase pump, and at specific ion channels e.g. L-type calcium channels^8,9^. Mg offers cell membrane stabilising properties which may help maintain sinus rhythm in "at risk" atria^9^.

Although hypomagnesaemia (serum Mg concentration < 0.6 mmol/L) is common in critical care, the evidence supporting supplementation is mixed^10^. The bulk of evidence comes from cardiac surgery, with the two most recent meta-analyses of existing Randomised Controlled Trials (RCTs) and a Cochrane Review finding supplemental Mg (or a higher serum Mg concentration) conveys a protective effect against developing AF^11–13^. This association has not been universally replicated in observational work, with several studies reporting the opposite relationship^14–16^. It is possible, that any inverse relationship seen in observational studies is a consequence of confounding by indication: that patients at greater risk of AF are more likely to receive supplementation.

Overall, outside the cardiac surgical population, there is little evidence supporting the routine supplementation of Mg for the purpose of preventing AF. Nevertheless, Mg administration, even at normal serum Mg concentrations, continues to form a routine part of critical care.

There are currently seven clinical trials of Mg for AF registered on clinicaltrials.gov (*June, 2021*). Of these, two are recruiting medical patients, the remainder focus on cardiac surgery. No studies address the question of the effectiveness of Mg supplementation for AF *prophylaxis*, instead focusing on its use as a treatment strategy.

Given the difficulties inherent to using observational methods to derive estimates of treatment effects, quasi-experimental techniques may offer potential solutions. Finding opportunities to conduct natural experiments can help limit the confounding effects of selection bias, and confounding by indication^17^. In particular, natural experiments based on clinician prescribing preferences as an instrumental variable, have been used to successfully investigate the efficacy of treatments such as anti-depressants, non-steroidal anti-inflammatories, and emergency general surgery^18–20^. In this setting, an instrumental variable is one which influences treatment exposure, whilst remaining otherwise unrelated to the outcome. For further information on instrumental variable analysis, Iwashyna and Kennedy have published a useful primer on the subject^21^.

In this study, we identified a natural experiment to evaluate the effect of Mg supplementation on developing AF. Specifically, that the bedside critical care nurse’s preference for Mg administration under varying conditions, may act as an instrumental variable to derive a more causally robust treatment effect estimate. We categorised individual nurses into ‘liberal’ or ‘restrictive’ Mg supplementation groups, based on their observed behaviour. That is, given the same measured serum Mg, are they more or less likely to supplement, relative to their colleagues in similar situations. Having dichotomised the nurse population according to prescribing preference, we then evaluated the relationship between patient exposure to liberal or restrictive nurse (the instrument) and the subsequent incidence of AF episodes. This permitted us to ascertain a more robust estimate of the association, by accounting for unmeasured confounding.

## Methods

### Ethics and data governance

This study used routinely collected Electronic Health Record (EHR) data from critical care admissions to a tertiary referral centre in the United Kingdom. Patient data was collected under the auspices of the Critical Care Health Informatics Collaborative (CCHIC). CCHIC has an exemption to collect identifiable clinical data under an opt-out consent framework, for the purposes of conducting secondary research. This is facilitated through an exemption to standard confidentiality law, detailed under Sect. 251 of the National Health Service (NHS) Act, 2006 and approved by the London—South East Research Ethics Committee (Ref: 19/LO/1017)^22^. An application for data access pertinent to the study question was approved by the CCHIC Scientific Advisory Group. Data was stored and analysed in the University College London Data Safe Haven, a secure data environment conforming to NHS Digital’s Information Governance Toolkit^22^.

### Study population

All adult admissions to University College London Hospital (UCLH) 35-bed general adult critical care unit between January 2016 and December 2017 were examined. These patients represent a mixed medical and surgical cohort, but no cardiac surgery is undertaken at our institution. Critical care patients have a daily serum Mg measurement taken, together with other routine blood tests. An “as required” prescription for supplemental Mg is available for the bedside nurse to access and administer as they see fit.

For the purposes of the study, each patient’s admission was divided into ‘observation windows’, equivalent to the nursing day shift. These windows consisted of a serum Mg measurement available at 8am, a subsequent opportunity for supplementation associated with the bedside critical care nurse, and a period of observation for AF prior to the next serum Mg measurement. Each patient therefore contributed an observation for as many days as they had serum Mg measured.

### Data preparation

In detail, each observation window starts with the measured serum Mg date-time stamp. Observation windows were censored by the earliest of the next serum Mg measurement, the end of the patient episode (discharge from critical care), or 24 h following initial measurement.

To reliably pair an individual critical care nurse with the observed serum Mg measurement, only serum Mg measurements recorded between midnight and 8 am were included. Thus, in the final data table, each row represents a serum Mg measurement which may be observed by the bedside nurse during the day shift. Similarly, Mg administrations occurring after 8 pm were removed as these would be associated with a different nurse.

All the recorded values for heart rhythm were joined to the relevant observation windows using date-time stamps. Heart rhythm data is recorded as 31 different categories within CCHIC. These were condensed to a binary indication of sinus rhythm or AF. The AF category comprised atrial flutter and atrial tachycardia.

Using the same process, covariates deemed to be clinically relevant, and which are routinely collected by CCHIC, were extracted and assigned to the relevant observation window. Included covariates and transformations are listed in Supplementary Table S1, with an example of the observation windows in Supplementary Table S2.

AF was defined as a change in documented heart rhythm from the sinus rhythm category to the AF category, occurring within the observation window. As part of the patient’s routine observations on the critical care unit, the bedside nurse inputs the current heart rhythm at a minimum hourly cadence from direct observation of the continuous cardiac monitor.

### Exclusion criteria

**Figure 1:**
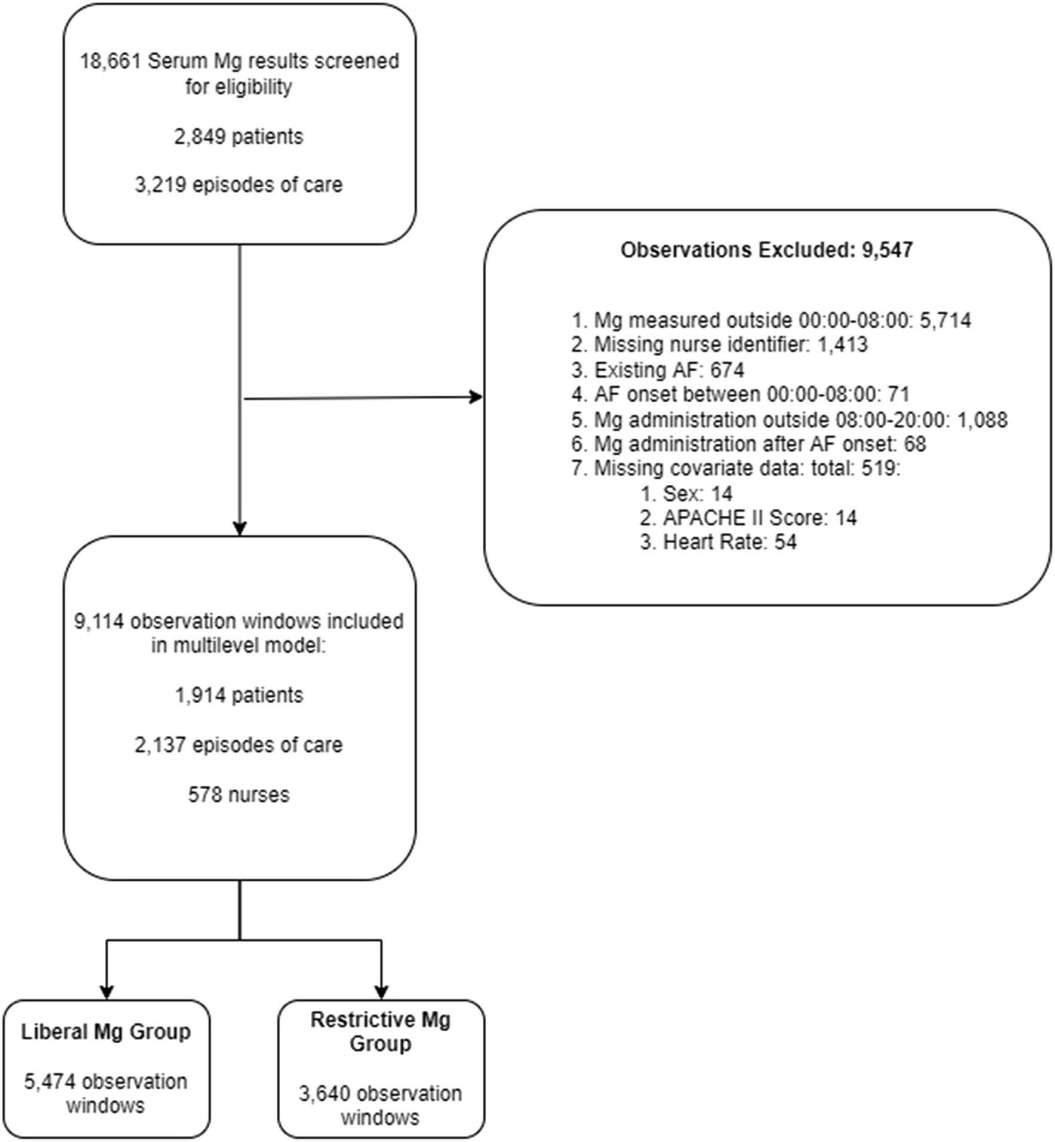
Study CONSORT diagram.

Observation windows were excluded from the analysis if the patient was already in atrial fibrillation (not new-onset), or where the Mg was administered after a AF event (indicating administration for treatment, rather than prophylaxis). As complete cases are required for an instrumental variable analysis, observation windows missing covariate data were excluded. Overall, this represented 3% (512) of the total number of observation windows examined. A breakdown of missing data is illustrated in Fig. 1. Infusions such as noradrenaline were assumed to be switched off rather than missing if infusion rate was not reported. This matches clinical reporting practice on the critical care unit.

### Statistical analysis

To identify the extent of variation in Mg supplementation practices and thus establish individual nurse preferences, we constructed a multilevel model to predict the likelihood of supplementation for each observation window. Windows were nested by nurse identity, which acted as a random effect in the model. Additional covariates which were deemed to be clinically relevant to the supplementation decision were added to the model as fixed effects. These included measured serum Mg, previous AF within the same critical care admission, noradrenaline dose, and illness severity (Acute Physiology and Chronic Health Evaluation II score). Individual covariates were added in a step-wise fashion to optimise inclusion of clinically important variables, minimise the model Akaike Information Criteria value, and maintain model convergence.

The predicted probabilities of Mg supplementation for each observation window, nested in individual critical care nurse identities were then used to conduct a prescribing preference instrumental variable analysis. In keeping with this design, it was assumed that the allocation of nurse to a particular patient on a particular day was both random in nature, and could not affect that patient’s chance of developing AF, other than through the nurse’s preference for supplementing Mg. A discussion of the assumptions required by the instrumental variable analysis is presented in the supplementary materials. Keele et al. present a detailed explanation of this method in their work on estimating the effect of emergency general surgery^20^.

Nurse identities were divided into ‘Liberal’ and ‘Restrictive’ Mg supplementation groups by splitting the random intercepts assigned to each nurse by the median intercept value obtained from the multilevel model. Following this, a two-stage least squares (2SLS) instrumental variable regression model was constructed to estimate the effect of Mg supplementation on AF occurrence within each observation window, using the nurse’s group assignment as the instrument. Clinically relevant covariates were then added. The instrumental variable model was tested using the weak instrument F test on the nurse group^23^, and the Wu-Hausman test for endogeneity^24^. To address potential violation of the 2SLS linearity assumption, a further instrumental variable model was constructed, using a Probit link to account for the binary outcome variable. We performed data cleaning, multilevel modelling and 2SLS regression in R^25^. We performed the instrumental variable Probit analysis in Stata^26^.

## Results

9114 observation windows were included in the analysis, representing 2137 critical care admissions for 1914 patients. Figure 1 illustrates participant flow through the study, with data loss at each stage. There were 578 nurses associated with the observation windows. Table 1 summarises the patient characteristics for the sample population. The mean serum Mg on admission to critical care was 0.94 mmol/L (SD 0.24 mmol/L). 55% (1057) of patients received at least one Mg supplementation during their admission. 5.38% (103) of patients had at least one documented episode of AF.

**Table 1:** Summary of study population.

Variable	Summary
Age (years)*	62 (24)
Sex *n* (%)	979 (51.2) female
Length of critical care stay (days)*	3.58 (5.08)
APACHE II score*	17 (9)
Serum potassium on admission (mmol/L)**	4.51 (0.56)
Serum magnesium on admission (mmol/L)**	0.97 (0.24)
Serum pH on admission**	7.39 (0.06)
At least one magnesium supplementation *n* (%)	1057 (55.22)
At least one atrial fibrillation event *n* (%)	103 (5.38)

*Non-normally distributed variables described using median (interquartile range).

**Normally distributed variables described using mean (standard deviation).

The first stage analysis constructed a multilevel model to estimate the probability of Mg supplementation in each observation window, nested by individual critical care nurse. The results from this model are included in Supplementary Table S3. After adjusting for measured serum Mg, previous AF within the same critical care admission, noradrenaline dose and illness severity, approximately 32% of variation in Mg supplementation observed in the model was attributable to the individual nurse.

Using the multilevel model, predicted probabilities for Mg supplementation within each observation window were obtained. These were used to divide nurses into ‘Liberal’ and ‘Restrictive’ supplementation groups for the instrumental variable analysis. 289 nurses were assigned to each group, corresponding to 5474 observation windows in the ‘Liberal’ group and 3640 in the ‘Restrictive’ group. Nurses in the ‘Liberal’ group observed a median of seven observation windows (IQR 32), and nurses in the ‘Restrictive’ group observed a median of three (IQR 17) observation windows.

Tables 2 and 3 illustrate the characteristics of individual patients, and observation windows (the unit of analysis), in both supplementation groups. Overall, both groups were similar for all the included covariates, but differed significantly in Mg supplementation (2640 administrations in the ‘Liberal’ group, versus 1027 administrations in the ‘Restrictive’ group). The incidence of AF was higher (78 episodes, 2.03%) in the ‘Restrictive’ supplementation group compared to the ‘Liberal’ group (74 episodes, 1.74%).

**Table 2:** Patient level characteristics of liberal and restrictive magnesium supplementation groups.

Variable	Liberal	Restrictive
Age (years), median (IQR)	62 (23)	61 (24)
Sex (%)	Female: 50	Female: 51
Length of stay (days), median (IQR)	4.4 (5.9)	4.7 (6.7)
APACHE II score, median (IQR)	17 (9)	17 (9)
Unit mortality (%)	10.9	11.8

**Table 3:** Observation window characteristics of liberal and restrictive magnesium supplementation groups.

Variable	Liberal (*n* = 5474)	Restrictive (*n* = 3640)
Mean (SD) serum potassium (mmol/L)	4.58 (0.50)	4.58 (0.51)
Mean (SD) serum magnesium (mmol/L)	0.94 (0.21)	0.95 (0.21)
Mean (SD) serum pH	7.40 (0.06)	7.40 (0.06)
Mean (SD) noradrenaline use (prev. 24 h) (mcg kg min^−1^)	0.02 (0.07)	0.02 (0.07)
Number of magnesium administrations (%)	2640 (48.2)	1027 (28.2)
Number of atrial fibrillation events (%)	78 (1.42)	74 (2.03)

The results of the instrumental variable regression estimating the association between Mg supplementation and AF are summarised in Table 4. After accounting for age, sex, illness severity (APACHE II Score), previous AF during the same critical care admission, baseline serum Mg, serum potassium, pH, heart rate, and mean noradrenaline administration, Mg supplementation was associated with a 3% decrease in relative risk of experiencing AF (95% CI − 0.06 to − 0.004, *p* = 0.03), an absolute risk reduction of 0.6%.

**Table 4:** Instrumental variable model estimates of the effect of magnesium supplementation on chance of experiencing an atrial fibrillation event.

Variable	Estimate	Standard error	*P* value
Intercept	0.028	0.007	< 0.001
Magnesium supplementation	− 0.033	0.015	0.028
Age (standardised)	0.01	0.002	< 0.001
Sex (male)	− 0.009	0.003	0.002
APACHE II score (standardised)	− 0.001	0.001	0.353
Previous atrial fibrillation	0.046	0.006	< 0.001
Serum magnesium (standardised)	− 0.003	0.004	0.492
Serum potassium (standardised)	0.0002	0.001	0.9
Heart rate (standardised)	0.0039	0.001	0.009
pH (standardised)	0.0016	0.001	0.284
Mean noradrenaline use (previous 24 h) (standardised)	0.0088	0.025	< 0.001

The weak instrument test indicated that nurse group was predictive of receiving Mg supplementation (*p* < 0.01), and was therefore not a weak instrument. The Wu-Hausman test suggested the presence of endogeneity in the Mg supplementation variable (*p* = 0.01), and therefore confirms the utility of conducting an instrumental variable analysis over logistic regression.

To ensure the use of 2SLS was not invalidated by the non-linearity of the outcome measure, we conducted a second regression using a Probit link function. The results are shown in Supplementary Table S4. After accounting for the same measured covariates, Mg supplementation continued to be associated with decreased AF (*p* = 0.01).

## Discussion

In this prescribing preference instrumental variable study, administering supplemental Mg was associated with a 3% decreased chance of developing AF, in a general critical care population, after adjusting for observed covariates.

In the context of the two most recent observational studies examining Mg supplementation in the post-cardiac surgery population, the results of this study are contrasting. Both studies found supplemental Mg to be associated with a higher chance of developing post-operative AF^14,15^. Instead, this study concurs with the experimental literature in the cardiac surgery population. In a 2019 systematic review and meta-analysis, Chaudhury et al. examined 2430 patients, across 20 RCTs of patients undergoing coronary artery bypass surgery^27^. Their results showed Mg supplementation was associated with a significant reduction in post-operative AF (RR 0.76; 95% CI 0.58–0.99; *p* = 0.04; I2 17.6%). This confirms previous work by Gu et al. in the same surgical cohort, but the level of study heterogeneity has been consistently high across all meta-analyses in this field^13^.

One explanation for the contrasting treatment effect directions seen across observational and experimental work is that the overall treatment effect of Mg supplementation is small, and as such is sensitive to bias. Another explanation of the results from Howitt and Lancaster is confounding by indication. It is possible that using an instrument to correct for unmeasured confounding was able to provide a more reliable estimate of treatment effect, in this case demonstrating that Mg supplementation reduces the risk of AF.

In addition to the identification of a more causally robust treatment effect estimate through use of a natural experiment, this study examines this problem in a general adult critical care population. Whilst the importance of AF is certainly highest in the post-cardiac surgery population, it is a negative complication in any critically ill patient and thus should be investigated and minimised through the application of evidence-based interventions.

This study has several limitations. It is a single centre study, and so has limited generalisability. Also, there was a large degree of missing data which precluded expanding the study beyond the stated time period. This illustrates some of the difficulties in conducting observational research using routinely collected EHR data. As systems mature and data collection becomes more automated, it is likely this problem will be minimised. Additionally, we were limited to data currently available in CCHIC. At the time of writing, data regarding past medical history, concurrent medication use (e.g. beta blockers), and reason for critical care admission was not available for inclusion. Work to expand the data set is ongoing.

We also included serial episodes of AF throughout a patient’s episode of care and therefore not just cases of new-onset AF. To explore this we conducted a sensitivity analysis using only first AF events within the data set. This analysis is described in Tables S5–S8 of the supplementary materials, but found a similar reduction in chance of developing AF having received magnesium supplementation.

The instrumental variable design itself requires several assumptions which bear mention. First, that levels of the exposure are all adequately represented in the data (stable unit treatment value assumption, SUTVA). Second, that that the instrument (critical care nurse) induces variation in the exposure (administration of Mg). Third, that the instrument may only determine the outcome through its action on the exposure. Fourth, that the instrument operates on an “as-if random” basis, and fifth, that there is no systematic non-compliance with the random nature of the instrument (monotonicity). Except for the second, which is evidenced in the first stage of the analysis, these assumptions are not easily objectively validated. Detailed discussions of the IV assumptions are included in the supplementary materials.

## Conclusions

This study has demonstrated the novel use of a natural experiment, using the critical care nurse’s prescribing preferences as an instrument to define a causally robust estimate of the treatment effect of supplemental Mg for the prophylaxis of AF, in a general critical care population. Clinically, this supports the continued administration of supplemental Mg in this context, but further work is necessary to define the optimal target serum Mg for supplementation and define effectiveness in populations with differing baseline risk. This study highlights the problem of continued use of poorly evidenced treatments in critical care medicine. Increasing availability of intelligent EHR systems may help to address these issues, through the efficient and scalable implementation of comparative effectiveness research studies comparing different routine treatment strategies across multiple subgroups.

## Supplementary Information

Supplementary Information.
